# Dysbiosis of intestinal microbiota in early life aggravates high-fat diet induced dysmetabolism in adult mice

**DOI:** 10.1186/s12866-021-02263-6

**Published:** 2021-07-08

**Authors:** Z. H. Miao, W. X. Zhou, R. Y. Cheng, H. J. Liang, F. L. Jiang, X. Shen, J. H. Lu, M. Li, F. He

**Affiliations:** 1 Department of Nutrition and Food Hygiene, West China School of Public Health and West China Fourth Hospital, Sichuan University, 610041 Chengdu, Sichuan P. R. China; 2 Basic Research and Development Center, Hebei Inatrual Bio-tech Co., Ltd, Shijiazhuang, Hebei P. R. China

**Keywords:** Early life, Intestinal microbiota, High-fat diet, Metabolism, Visceral obesity

## Abstract

**Background:**

Accumulating evidence have shown that the intestinal microbiota plays an important role in prevention of host obesity and metabolism disorders. Recent studies also demonstrate that early life is the key time for the colonization of intestinal microbes in host. However, there are few studies focusing on possible association between intestinal microbiota in the early life and metabolism in adulthood. Therefore the present study was conducted to examine whether the short term antibiotic and/or probiotic exposure in early life could affect intestinal microbes and their possible long term effects on host metabolism.

**Results:**

A high-fat diet resulted in glucose and lipid metabolism disorders with higher levels of visceral fat rate, insulin-resistance indices, and leptin. Exposure to ceftriaxone in early life aggravated the negative influences of a high-fat diet on mouse physiology. Orally fed TMC3115 protected mice, especially those who had received treatment throughout the whole study, from damage due to a high-fat diet, such as increases in levels of fasting blood glucose and serum levels of insulin, leptin, and IR indices. Exposure to ceftriaxone during the first 2 weeks of life was linked to dysbiosis of the fecal microbiota with a significant decrease in the species richness and diversity. However, the influence of orally fed ceftriaxone on the fecal microbiota was limited to 12 weeks after the termination of treatment. Of note, at week 12 there were still some differences in the composition of intestinal microbiota between mice provided with high fat diet and antibiotic exposure and those only fed a high fat diet.

**Conclusions:**

These results indicated that exposure to antibiotics, such as ceftriaxone, in early life may aggravate the negative influences of a high-fat diet on the physiology of the host animal. These results also suggest that the crosstalk between the host and their intestinal microbiota in early life may be more important than that in adulthood, even though the same intestinal microbes are present in adulthood.

**Supplementary Information:**

The online version contains supplementary material available at 10.1186/s12866-021-02263-6.

## Background

Obesity is regarded as a worldwide public health issue, and the Global Burden of Disease study shows that the prevalence of obesity has more than doubled since 1980 [1]. Widely published studies have identified obesity as a major risk factor for various noncommunicable diseases (NCD) [2, 3], including diabetes, hypertension, dyslipidemia, insulin resistance, and cardiovascular diseases [4, 5]. Importantly, early life, especially the first 1000 days, is regarded as the key period for infant growth and has long-term effects even on adulthood health and diseases [6]. Mounting evidence has identified that the intestinal microbiota in early life has strong correlations with NCD during adulthood, including obesity, but the underlying mechanisms and possible preventative treatments remain unclear [7, 8].

The intestinal microbiota has been widely proven to contribute to human health [9, 10]. Therefore, the colonization of the intestinal microbiota during early development is important for host health. Previous studies have shown that there were significant differences between the intestinal microbiota of infants and adults and that infants were more sensitive to environmental factors than adults, suggesting that early life may be of key importance to the establishment of the intestinal microbiota [11]. Our previous study also indicated that early life is the key time for the formation of intestinal microbiota in infants [12]. Therefore, research on the association between the formation of the intestinal microbiota in infancy and long-term effects on adulthood health and diseases may contribute to the prevention of obesity in adulthood.

Antibiotics have been used to protect humans from severe infections and can be beneficial to patient health for extended periods. However, recent studies have indicated that antibiotics can alter the intestinal microbiota of patients [13]. Neonatal mice who received antibiotic treatment had a higher ratio of *Firmicutes* species to *Bacteroides* species–a key bacterial indicator for obesity–and lower alpha diversity, which is considered harmful to host health [14, 15]*.* Evidence also suggests that antibiotic use can alter the composition of the microbial community, and patients who had been treated with broad-spectrum antibiotics were found to be more consistently associated with overweight or obesity symptoms [16, 17]. Therefore, the dysbiosis of the intestinal microbiota may be an underlying mechanism whereby antibiotic use leads to obesity and metabolic disorders.

Probiotics are defined as “live microorganisms” that “when administered in adequate amounts, confer a health benefit to the host” and are regarded as functional foods with protective effects against obesity and metabolic diseases [18]. Recent studies have demonstrated that supplementation with several probiotics may have beneficial effects on host metabolism, regardless of whether these are humans or animals [19, 20]. An analysis of the whole genome of *Bifidobacterium bifidum* TMC3115 (TMC3115) isolated from healthy infants revealed encoded loci for the utilization of human milk oligosaccharides. These studies have indicated that the abnormal intestinal microbiota induced by antibiotic treatment in early life could impair the epithelium and affect immunity through to adulthood and that TMC3115 may alleviate the side effects caused by ceftriaxone [21]. However, it remains unclear whether TMC3115 could mitigate the metabolic disorders induced by the dysbiosis of the intestinal microbiota.

Our previous study identified that short-term use of ceftriaxone can damage the intestinal microbiota in young mice [22]. Therefore, this study was conducted to determine whether the short-term antibiotic and/or probiotic exposure in early life could affect the construction of the intestinal microbiota and the possible long-term effects of these treatments on host metabolism.

## Results

### Body weight and visceral fat rate

**Fig. 1:**
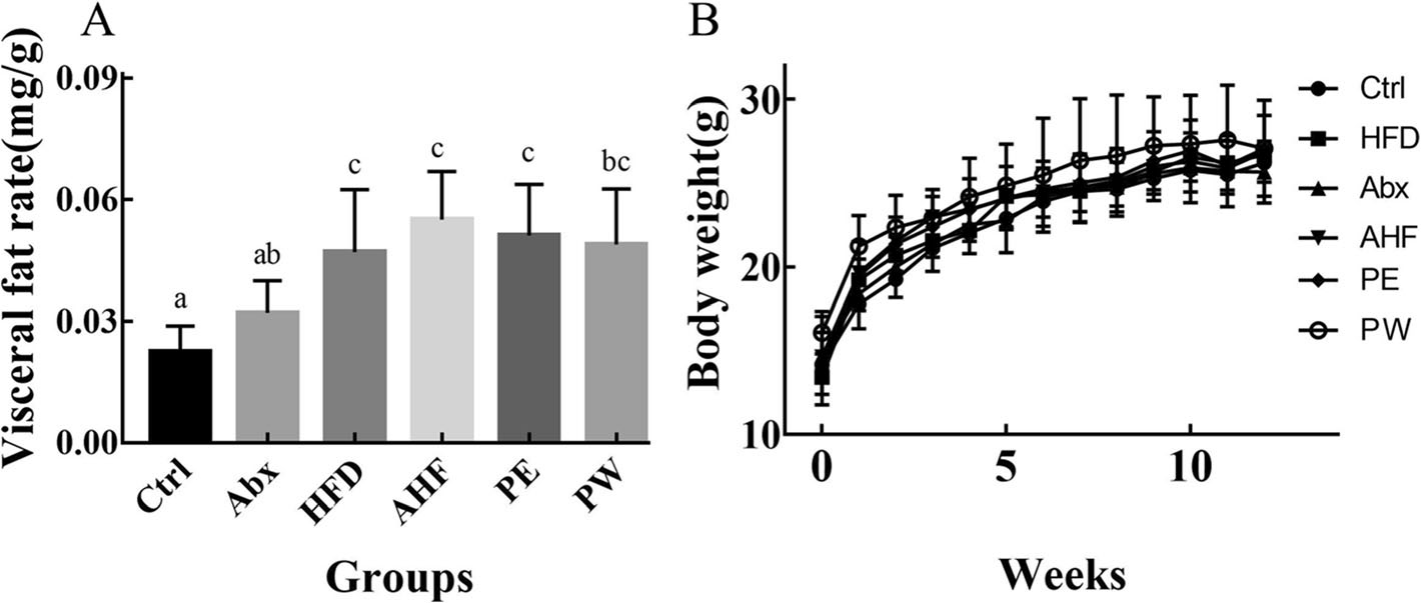
The result of body weight and visceral fat rate (*n* = 12/group). Values are expressed as mean ± SEM (**a**) The visceral fat rate of mice in different groups. (**b**) The body weight of mice in different groups. There were no significant differences among groups with a common letter *P* < 0.05

Although there was no significant difference between mouse body weights (Fig. 1), the visceral fat rate (total visceral fat/body weight) was significantly different among the six groups (*p* < 0.05). Providing mice with a high-fat diet resulted in a higher visceral fat rate relative to the normal diet group (*p* < 0.05). However, probiotic treatment (both PE and PW groups) did not reduce the visceral fat rate of mice compared with that of the AHF group (*p* < 0.05) (Fig. 1).

### Impairment of FBG and OGTT

**Fig. 2:**
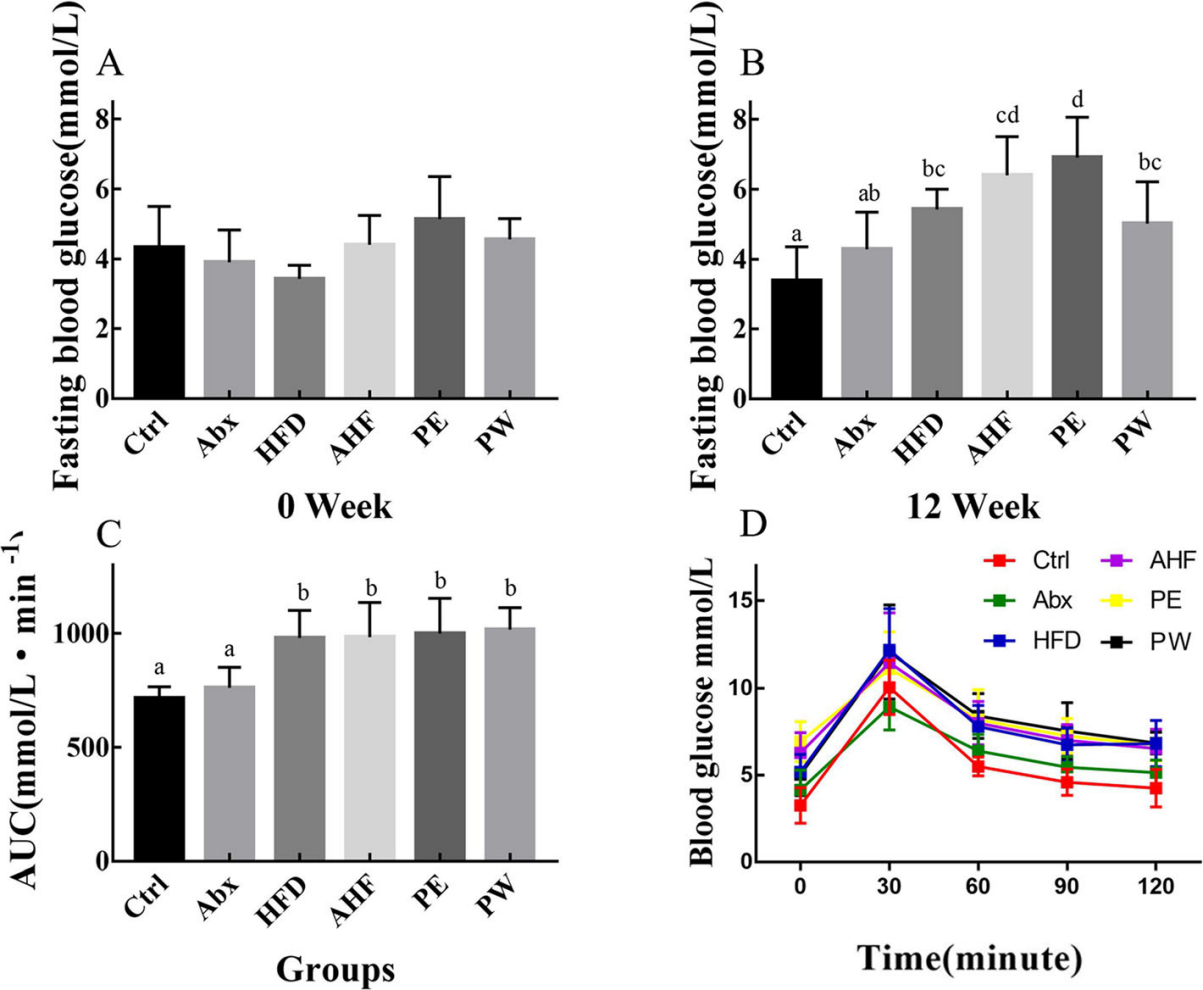
The result of fasting blood glucose and oral glucose tolerance test (*n =* 12/group). Values are expressed as mean ± SEM (**a**) The fasting blood glucose of mice at week 0. (**b**) The fasting blood glucose of mice at week 12. (**c**) The area under the curve values of mice in different groups. (**d**) The oral glucose tolerance test of mice in different groups. There were no significant differences among groups with a common letter *P* < 0.05

At the end of the antibiotic treatment (week 0), the level of FBG in mice in the test groups was not significantly different from that in mice in the Ctrl group (Fig. 2). Mice fed with a high-fat diet had significantly higher FBG levels and higher AUC values compared with those of the Ctrl group mice (*p* < 0.05). Additionally, at week 12, there was an increasing trend in the FBG levels of mice in the AHF group compared with the HFD group, whereas the PW group displayed a decreasing trend of FBG levels compared with the AHF group (Fig. 2). However, the AUC values did not show any differences among mice fed with a high-fat diet.

### Level of serum and liver lipid metabolism

**Fig. 3:**
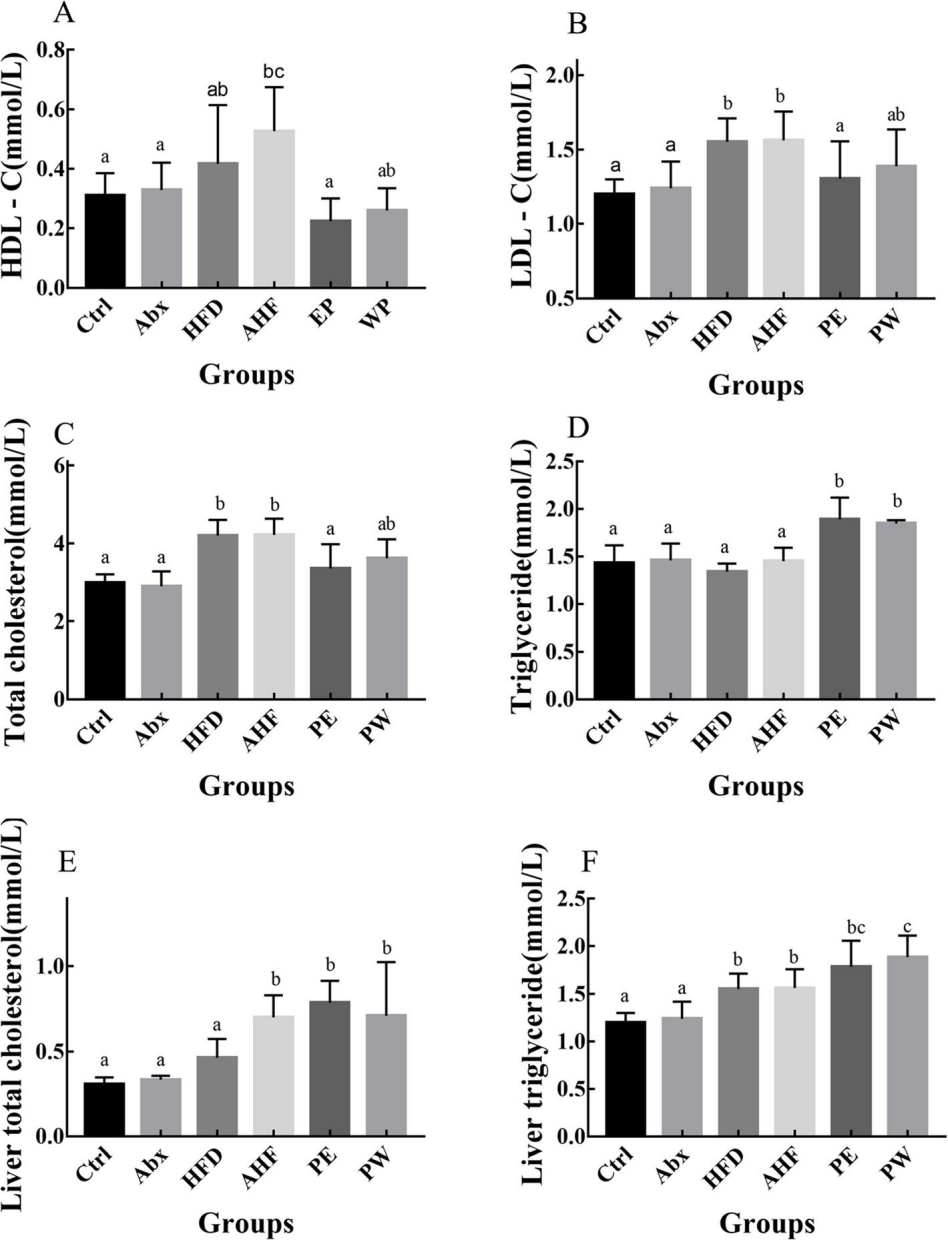
The lipid metabolism-related index in mice (*n =* 12/group). Values are expressed as mean ± SEM (**a**) Plasma levels of high-density lipoprotein cholesterol. (**b**) Plasma levels of low-density lipoprotein cholesterol. (**c**) Plasma levels of total cholesterol. (**d**) Plasma levels of triglyceride (**e**) Liver levels of total cholesterol. (**f**) Liver levels of triglyceride. There were no significant differences among groups with a common letter *P* < 0.05

No significant differences were observed in the serum levels of HDL-c among the six groups. However, when compared with the Ctrl group, mice in the HFD and AHF groups had increased levels of LDL-c and serum total cholesterol (*p* < 0.05), although this was alleviated by TMC3115 treatment (Fig. 3). In addition, a high-fat diet lead to higher levels of liver triglyceride and liver total cholesterol (*p* < 0.05), and these were not affected by probiotic treatment (Fig. 3).

### Serum level of insulin, adiponectin, and leptin

**Fig. 4:**
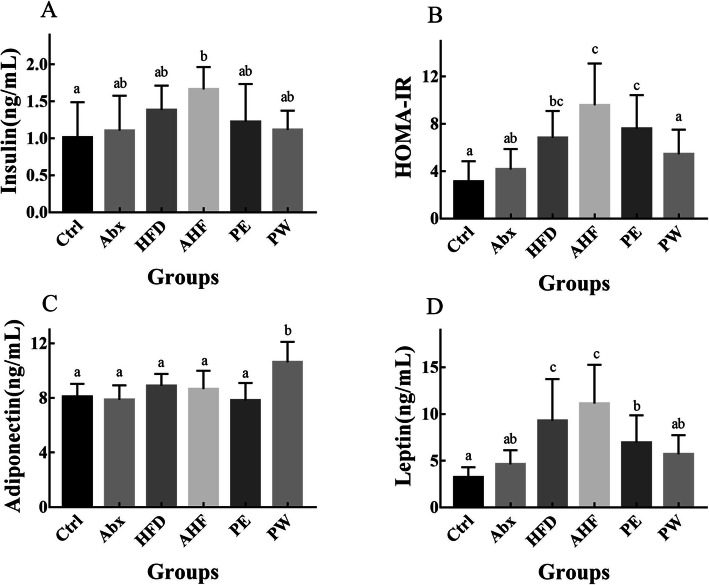
The result of metabolism-related hormones and insulin resistance (*n =* 12/group). Values are expressed as mean ± SEM (**a**) The serum level of insulin (**b**) The insulin-resistance indices of mice in different groups. (**c**) The serum level of adiponectin. (**d**) The serum level of leptin. There were no significant differences among groups with a common letter *P* < 0.05

Although mice in the HFD group did not have higher serum insulin levels compared with those in the Ctrl group, mice in the AHF group did have significantly higher levels of serum insulin compared with mice in the Ctrl group (*p* < 0.05) (Fig. 4). Meanwhile, there was a decreasing trend of serum insulin levels in the PW group compared with the HFD and AHF groups. Mice fed a high-fat diet had higher levels of leptin and IR compared to mice provided with normal diet (*p* < 0.05), and antibiotic exposure resulted in an increasing trend of leptin and IR levels, both in mice in the normal diet group and in the high-fat diet group. Importantly, significantly lower levels of leptin and IR (*p* < 0.05) and a higher level of adiponectin were observed in the PW group compared with the AHF group (Fig. 4).

### Fecal microbiota analysis

**Fig. 5:**
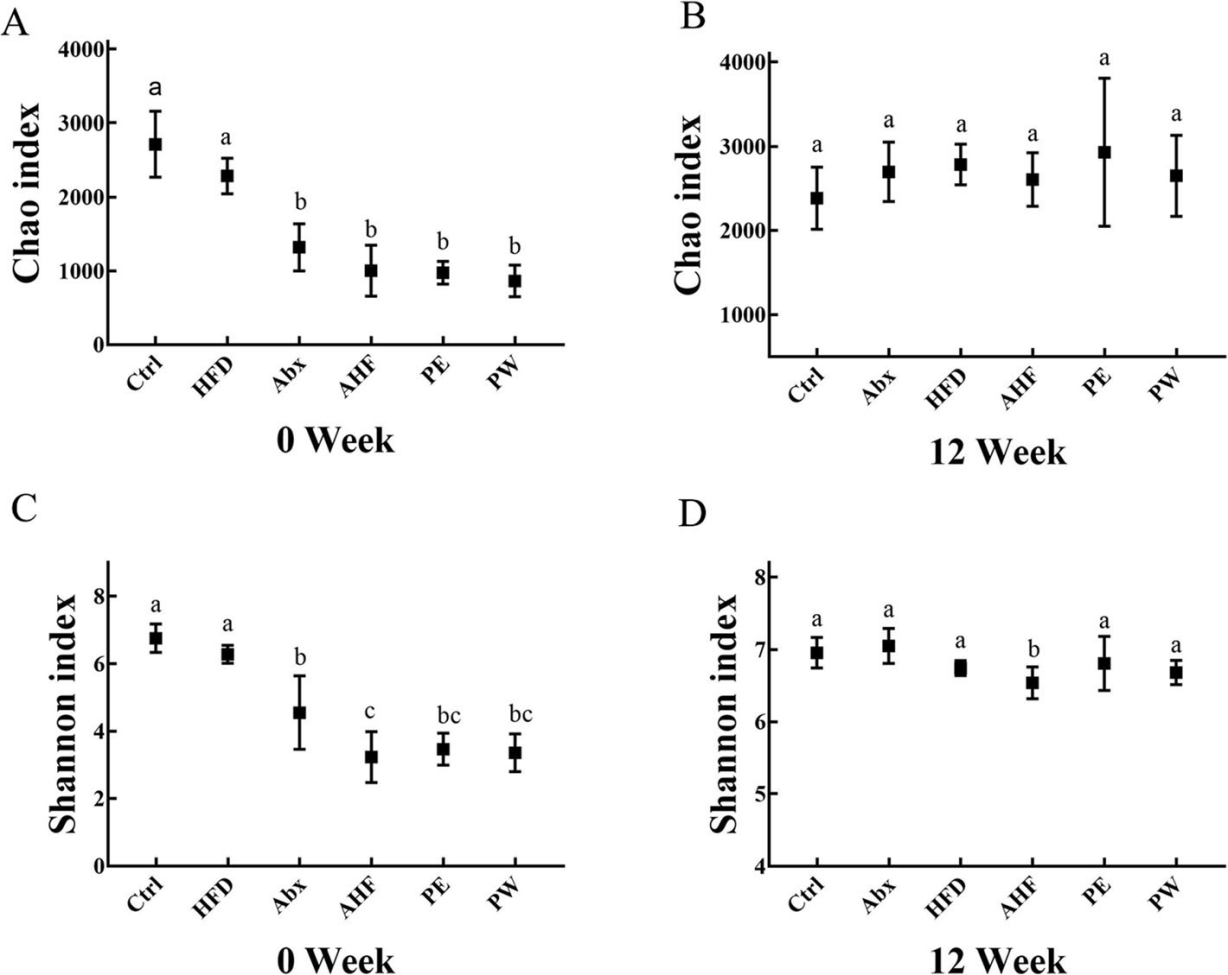
The α-diversity of intestinal microbiota. (**a**) The Chao indices of intestinal microbiota at week 0 (**b**) The Chao indices of intestinal microbiota at week 12. (**c**) The Shannon indices of intestinal microbiota at week 0. (**d**) The Shannon indices of intestinal microbiota at week 12

**Fig. 6:**
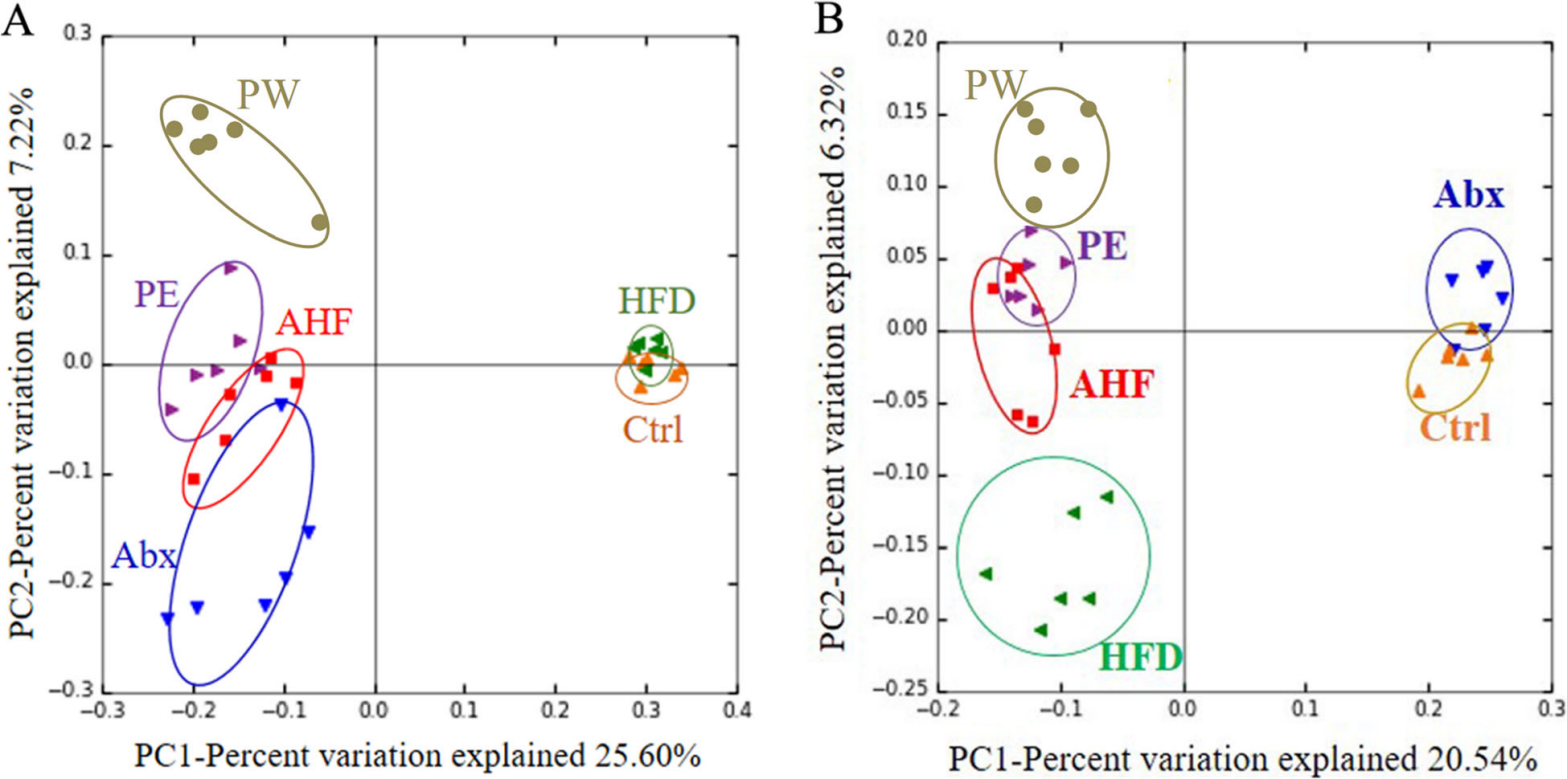
The β-diversity of of intestinal microbiota. (**a**) Principal coordinate analysis based on the unweighted UniFrac distance of operational taxonomic units at week 0. (**b**) Principal coordinate analysis based on the unweighted UniFrac distance of operational taxonomic units at week 12

After gavage, the Chao and Shannon indices both significantly decrease in mice treated with an antibiotic (*p* < 0.05) (Fig. 5). After 12 weeks, only the Shannon indices of the AHF group exhibited a significant decrease compared with that of the Ctrl and Abx groups (Fig. 5). PCoA analysis based on unweighted UniFrac distance was used to reflect the construction of the intestinal microbiota and demonstrated that the intestinal microbiota composition of each group formed a significant cluster(Fig. 6). At week 0, principal component (PC)1 and PC2 explained 25.60 and 7.22% of variability, respectively, and after 12 weeks, PC1 and PC2 explained 20.54 and 6.32% of variability, respectively.

**Fig. 7:**
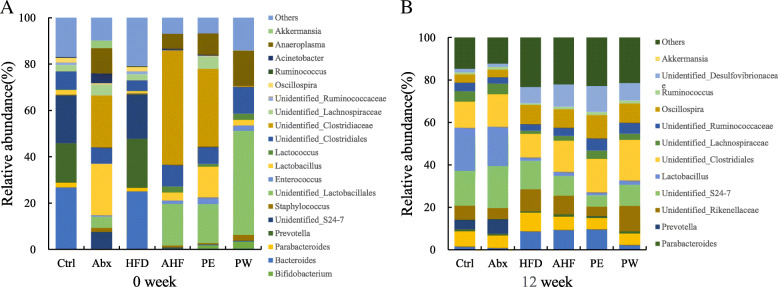
Effects of antibiotic/probiotic treatment and high fat diet on intestinal microbiota. (**a**) Relative abundance at the phylum level at week 0 (**b**) Relative abundance at the phylum level at week 12. (**c**) Relative abundance at the genus level at week 0. (**d**) Relative abundance at the genus level at week 12

At the phylum level, the relative abundance of *Firmicutes* increased sharply, whereas that of *Bacteroidetes* decreased significantly in the Abx/AHF/PE/PW groups because of antibiotic treatment at week 0 (Fig. 7). Notably, the relative abundance of *Actinobacteria* included *Bifidobacterium* TMC3115 and was observed to increase significantly in both the PE and PW groups. After 12 weeks, the Abx group exhibited a similar species abundance at the phylum level compared with that of the Ctrl group. In addition, there was a significant increase of *Proteobacteria* in mice fed with a high-fat diet. Meanwhile, the relative abundance of *Proteobacteria* increased in the AHF group compared with the HFD group. At the genus level, antibiotic treatment led to a significant decrease of *Parabacteroides*, *Prevotella*, *Ruminococcus*, *Oscillospira*, and *Bacteroides* genera after antibiotic treatment (Fig. 7). Additionally, the PE and PW groups had a higher abundance of *Bifidobacterium* species. After 12 weeks, a high-fat diet resulted in a significant decrease of *Prevotella*, *Ruminococcus*, *Bifidobacterium*, and *Ruminococcaceae* species. Then, as expected, the abundance of *Bifidobacterium* species in the groups treated with TMC3115, especially in the PW group, significantly increased.

## Discussion

A high-fat diet is known to potentially result in obesity, and long-term microbiome dysbiosis induced by antibiotic treatment can lead to further weight gain and higher fat mass in mice fed with HFD [23]. Female BALB/c mice fed with a 12-week high-fat diet developed higher visceral obesity, although there were no significant differences in body weight among the tested mice, which may be explained by the strain, sex, and food intake of mice [24]. Additionally, the mice treated with ceftriaxone had a higher trend of visceral fat rate, and the same trend was observed between mice fed a HFD treated with or without ceftriaxone. These results indicate that antibiotic treatment, even in early life, may promote a higher deposition of visceral fat caused by a high-fat diet. In our previous studies, TMC3115 was proven to help in alleviating the dysbiosis of the intestinal microbiota induced by antibiotics [21]. In this study, although there was no significant difference in the visceral obesity of mice treated with TMC3115 and those without TMC3115, the long-term use of TMC3115 did show a decreased trend in visceral obesity. Thus, our results suggest that exposure to antibiotics in infancy may influence the accumulation of visceral fat in the host during adulthood and that the presence of TMC3115 may mitigate these effects.

The FBG levels and AUC values in OGTT were determined to assess the effects of HFD and antibiotic/probiotic treatment in the glucose metabolism of mice. Previous studies have shown that a high-fat diet could result in higher FBG levels and AUC values [25]. In this study, at week 0, there were no significant differences among the tested mice, whereas at week 12, mice fed a high-fat diet all had higher FBG levels than mice fed a normal diet. Additionally, recent research has shown that probiotic treatment, especially with *Bifidobacterium* species, may contribute to FBG levels and AUC values [26, 27]. Our results also demonstrated that TMC3115 was associated with a decreased trend in FBG levels in mice but did not improve the AUC values. Mice exposed to antibiotics and fed with a high-fat diet had an increased trend in FBG levels compared with mice exposed to ceftriaxone alone, whereas those treated with TMC3115 only had a decreasing trend in FBG levels. Therefore, these results indicate that the dysbiosis of the intestinal microbiota in early life can aggravate the dysmetabolism of the host in adulthood. Meanwhile, the long-term use of TMC3115 may partly resist the effects induced by antibiotic treatment in early life.

Similarly to other studies, a high-fat diet significantly increased TC and LDL-C in the tested mice without influencing the TG level [28, 29]. A high-fat diet also led to higher levels of liver TG and liver TC than mice fed a normal diet. Antibiotic treatment resulted in a further elevation of liver TC levels in mice fed with a high-fat diet. Although previous studies have reported that a number of *Bifidobacterium* species can improve dyslipidemia caused by a high-fat diet, this was not the case with TMC3115. However, considering that the long-term use of TMC3115 was associated with a decreasing trend of visceral obesity, these results may be explained by the use of TMC3115 to inhibit the absorption of serum lipids, further affect lipid metabolism, and alleviate the accumulation of visceral fat. Therefore, these results may demonstrate that exposure to antibiotics in early life may promote lipid dysmetabolism in adulthood and that TMC3115 may alter the lipid dysmetabolism induced by a high-fat diet in the host.

The serum levels of insulin, leptin, and adiponectin are known to be closely related to host metabolism [30, 31]. For instance, adiponectin can improve IR by decreasing the muscular lipid contents in mice, and leptin can promote energy expenditure by increasing thyroid hormone signaling [32, 33]. Antibiotic treatment in early life also led to a higher trend of leptin levels compared with those of mice fed on the same diet. The use of TMC3115 in early life significantly decreased the levels of leptin, and the long-term use of TMC3115 significantly increased the levels of adiponectin. Although research has reported that a high-fat diet can increase insulin levels and decrease adiponectin levels, there were no significant differences in adiponectin levels between mice fed with a normal diet and those fed with a high-fat diet [34]. Previous studies have demonstrated that the sensitivity of adiponectin levels to a high-fat diet in female mice was different compared with those in male mice, which may explain the results of serum adiponectin in our studies [35, 36].

A high-fat diet can also result in a higher level of insulin and HOMA-IR [37, 38]. Similarly, the results from the present study also showed that a high-fat diet led to an increased trend in insulin and IR levels. Moreover, antibiotic treatment in early life further impaired the insulin levels and HOMA-IR in mice fed with a high-fat diet. In contrast to the effects on mice treated with antibiotics, even the use of TMC3115 in early life led to a decreased trend in insulin and IR levels. In addition, the long-term use of TMC3115 showed a further decreased trend in serum insulin levels and a significant decrease in IR compared with those in mice in the PE group. These results further indicated that antibiotic treatment in early life might aggravate the dysmetabolism of the host with an unhealthy diet in adulthood, whereas TMC3115 could partly alter this effect.

The intestinal microbiota is a key factor that influences host health, including immunity, metabolism, and even neurobehavioral traits [39, 40]. One of those underlying mechanisms may be the metabolites of intestinal microbiota, such as short-chain fatty acids or bile acids that have been shown to influence host health. The short-chain fatty acids fermented by the intestinal microbiota can regulate energy uptake and secretion of hormones, such as peptide YY (PYY) and glucagon-like peptide 1 (GLP-1), by activating G protein-coupled receptor 43 (GPR 43) and G protein-coupled receptor 41 (GPR 41) [41, 42]. Meanwhile, the intestinal microbiota can also affect bile acids that regulate host metabolic pathways via the farnesoid X receptor (FXR) and G protein-coupled membrane receptor 5 (TGR 5) [43, 44]. Furthermore, previous studies also indicated that the balance of the intestinal microbiota community composition, especially that of *Firmicutes* and *Bacteroidetes*, might play an important role in the metabolism of host [15, 45]. In this study, it was clear that antibiotic treatment dramatically altered the intestinal microbiota composition immediately, but the effects induced by a short-term therapeutic dose of ceftriaxone on intestinal microbiota at least at the phylum level did not appear to continue to adulthood. Recently, research has explored the relationship between obesity and antibiotic treatment, especially in infancy or childhood, but the results lacked consistency [46–48]. In our study, antibiotic use induced the dysbiosis of the intestinal microbiota during early life, although this effect did not continue to adulthood. However, antibiotic treatment in early life increased the sensitivity of the host animal to a high-fat diet and enhanced the negative effects of a high-fat diet on host metabolism, although the alpha-diversity and beta-diversity of intestinal microbiota seemed to recover in adulthood after the termination of the antibiotic treatment. Thus, these results indicate that exposure to antibiotics in early life might damage or alter the physiological function and metabolism of the host animal in a complex and incurable manner. Moreover, these results indicate that the crosstalk between the host and their intestinal microbiota in early life might be more important than in adulthood, even with the same intestinal microbes. These results could also provide possible reasons for the differences among recent epidemiological investigations concerning the association between antibiotic treatment and obesity as the timing of the antibiotic treatment may be critical.

*Proteobacteria* have a relatively low abundance at the phylum level in a healthy host. However, previous studies have shown that the relative abundance of *Proteobacteria* in patients with obesity was significantly higher [49]. Further research also indicated that abnormal inflammation, obesity, and insulin resistance were related to a lower relative abundance of *Proteobacteria*, especially that of *Enterobacteriaceae* and *Desulfovibrionaceae* genera [50]*.* In this study, the relative abundance of *Proteobacteria* in mice fed with a high-fat diet was significantly higher than mice fed a normal diet, and treatment with antibiotics in early life resulted in an increased trend of *Proteobacteria* abundance. However, probiotic treatment did not alter the relative abundance of *Proteobacteria*. In addition, *Parabacteroides* species are known to contribute to alleviation of obesity and obesity-related dysfunctions in mice [51], whereas *Prevotella*, *Ruminococcus*, and *Bacteroides* species were considered to be beneficial to host health [52]. Previous studies have demonstrated that perturbations caused by short-term antibiotic treatment can recovery to baseline spontaneously [53]. In our study, antibiotic exposure in early life did lead to a significant decrease of these beneficial microbes at week 0, but the abundance of these species returned to their normal level after the 12 weeks normal diet. Importantly, the high-fat diet did decrease the abundance of *Prevotella*, *Parabacteroides*, and *Ruminococcus* genera. Although TMC3115 did not decrease the abundance of these genera, the abundance of *Bifidobacterium* species was increased. These results suggest that the crosstalk between the intestinal microbes and host may be timing dependent and that several microbial genera may have more impact on the host in early life.

Our study used the Chao indices (reflecting species richness) and Shannon indices (reflecting species diversity) to assess the alpha diversity of intestinal microbiota. Previous studies have demonstrated that lower alpha diversity was related to various diseases such as nonalcoholic fatty liver disease and obesity [54, 55]. Here, antibiotic treatment significantly decreased the Chao and Shannon indices in early life, whereas these influences did not last to adulthood. After week 12, no differences were observed in the Chao indices among the tested mice, a high-fat diet did lead to a decreasing trend in the Shannon indices, and antibiotic treatment resulted in a further decrease of the Shannon indices. The results of the UniFrac-based principal coordinate analysis showed that the short-term use of antibiotics in early life can dramatically alter the composition of the intestinal microbiota. After 12 weeks of antibiotic treatment, mice that had been administered solely with antibiotic by gavage had the most similarity in the composition of the intestinal microbiota compared with mice in other groups, whereas those fed with a high-fat diet had a significantly different composition of intestinal microbiota. Furthermore, mice treated with a high-fat diet and antibiotic in early life had a varied composition of intestinal microbiota compared with mice fed with a high-fat diet. These results indicated that the unhealthy composition of the intestinal microbiota induced by ceftriaxone in early life could promote intestinal microbiota disorders in the host later induced by a high-fat diet. By contrast, probiotic treatment with TMC3115, especially long term, could significantly alleviate these effects.

## Conclusion

In conclusion, these results demonstrated that exposure to antibiotics in infancy can influence the long-term health of host, for instance, in the accumulation of visceral fat, changes to the glycolipid metabolism, and levels of several related hormones, although the dysbiosis of the intestinal microbiota had mostly recovered in adulthood. Therefore, the crosstalk between the intestinal microbes and host animal may be timing dependent, and the host early life may be the key time for intestinal microbes to affect physical function and metabolism. Furthermore, the results from our study suggest that probiotic treatment might be used as a complementary strategy to protect people from damage caused by the dysbiosis of the intestinal microbiota and unhealthy diet habits.

## Methods

### Mice

Two-week-old female BALB/c mice from Chengdu Dossy Experimental Animals Co., Ltd. (Chengdu, China), were divided into (*n* = 12) the control group (Ctrl), antibiotic exposure group (Abx), high-fat diet group (HFD), antibiotic exposure + high-fat diet group (AHF), probiotic used in early life group (PE), and probiotic used throughout whole life group (PW). A specific pathogen-free facility with a 12-h light/dark cycle was used at an ambient temperature of 23 °C ± 3 °C and humidity of 40–70%. This study was approved by the Experimental Animal Management Committee of Sichuan Government (Approval number: SYXK2013–011).

At the end of the study, all mice were anesthetized by intraperitoneal injection of 2,2,2- tribromoethanol (125 mg/kg, CAS:75–80-9 Sigma-Aldrich Chemie GmbH, Steinheim, Germany) to collect blood samples via heart puncture. Then mice were euthanized by injection of an overdose of 2,2,2- tribromoethanol (500 mg/kg) and decapitated.

### Experimental schedule

The mouse experimental schedule is shown in Fig. S1. In the first 2 weeks (week − 2 to week 0), the Abx/AHF/PE/PW groups were treated with 0.2 mL (100 mg/kg) of ceftriaxone (Shanghai Aladdin Bio-Chem Technology Co., Ltd., Shanghai, China) once a day, and the Ctrl/HFD group received an equal volume of sterile saline. Meanwhile, the PE/PW groups were treated with 0.2 mL of 5 × 10^9^ CFU/mL TMC3115 (Hebei Inatural Biotech Co., Ltd., Hebei, China) after 2 h of antibiotic treatment, whereas mice in the other groups were administered an equal volume of sterile saline by gavage. For the next 12 weeks (week 0 to week 12), the PW group was treated with 0.2 mL of 5 × 10^9^ CFU/mL TMC3115 once a day, whereas the other groups received an equal volume of sterile saline.

All mice received a normal diet in early life (week − 2 to week 0). Then, the Ctrl/Abx group continued received a normal diet, and the HFD/AHF/PE/PW group received a high-fat diet (Research Diet D12492; Research Diets, New Brunswick, NJ, USA) for 12 weeks (week 0 to week 12).

### Fasting blood glucose(FBG) and oral glucose tolerance test (OGTT)

All mice were fasted from 9:00 p.m. to 9:00 a.m., and blood glucose levels were determined using a portable glucometer (ACCU-CHEK) at 0 and 12 weeks. At the end of week 12, after a 12 h fast, all mice were orally dosed with a glucose solution (2 g/kg). Blood glucose levels were determined using the portable glucometer at 0, 30, 60, 90, and 120 min after oral administration, and area under the curve (AUC) values were used to estimate the extent of glucose tolerance impairment.

### Serum and liver tissue supernatant analysis

Mouse blood was centrifuged at 2000×*g* for 20 min. Then, the serum was collected and was centrifuged again at 2000×*g* for 5 min. The serum level of insulin was determined using an enzyme-linked immunoassay (ELISA) kit (Merck Millipore, Burlington, MA, USA), and the insulin resistance indices(IR) was calculated by (FBG × fasting insulin)/22.5. The serum levels of adiponectin and leptin were measured using their respective ELISA kits (R&D Systems Inc., Minneapolis, MN, USA).

An Automatic Chemistry Analyzer-Chemray240 (Shenzhen Rayto Life and Analytical Sciences Co., Ltd.) was used to determine the serum level of triglyceride (TG), total cholesterol (TC), high-density lipoprotein (HDL), and low-density lipoprotein (LDL).

The levels of TG and TC in the liver were determined using commercially available kits: tissue total cholesterol assay kit-E1015 and tissue triglyceride assay kit-E1013 (Applygen Technologies Inc., Beijing, China).

### Fecal microbiota community determination by 16S rRNA sequencing

According to previous methods, the 16S rRNA sequencing was performed as follow [56]. The Qubit fluorometer (Life Technologies) and the TapeStation (Agilent) were used to concentrate and purify DNA and the 16S library was constructed in strict accordance recommended by Illumina. The V3-V4 region of the 16S rRNA gene was amplified using 341F and 806R fusion primers containing identification indexes. PCR was performed on a TaKaRa Cycler Dice Touch (TaKaRa) with 2 × KAPA HiFi HotStart ReadyMix (Kapa Biosystems Inc., USA) under the following conditions: initial denaturation at 95 °C for 3 min, followed by 25 cycles of 95 °C for 30 s, 55 °C for 30 s, and 72 °C for 30 s, and ended with an extension step at 72 °C for 5 min. Qubit fluorometer and TapeStation were used to analyze the DNA concentration and size. The AMPure XP magnetic beads (Beckman Coulter Inc., USA) were used to purify the above PCR products and these products were diluted and pooled. Next the Nextera XT Index Kit (Illumina, United States) was used to add the barcodes into the above pooled PCR products and the MiSeq Reagent Kit v3 (600-cycle) (Illumina) was used to purify and pool the indexed PCR products. USEARCH (v 6.1.544) software was used to remove the chimeric check and singleton and the low-quality sequences were filtered by default 0.97. The QIIME (v 1.9.1) pipeline was used to identify representative sequences for each operational taxonomic unit (OTU) and the GreenGenes 13.8 database was used to align them. Further processions of OTU tables were completed at genus and phylum levels.

The QIIME script core_diversity_analyses.py was used to perform the calculation of the Alpha and beta diversity of gut microbiota and the principal coordinate analysis plots (PCoA) based on unweighted UniFrac was used to calculate Beta diversity.

### Statistical analysis

All statistical analyses, except the results of the 16S rRNA sequencing, were performed using GraphPad Prism 7.0 software (GraphPad Software Inc., San Diego, CA, USA). The Shapiro–Wilk normality test was used to assess the normality. The Holm–Sidak’s multiple comparisons test or Kruskal–Wallis test was used for multiple comparisons. A *p*-value ≤0.05 was considered statistically significant.

## Supplementary Information

**Additional file 1.**

## Data Availability

Raw reads of 16S rRNA sequencing data is submitted to Sequence Read Archive (SRA) under the BioProject ID PRJNA636965 in NCBI.

## Abbreviations

IR: Insulin resistance indices
FBG: Fasting blood glucose:
TMC3115: *Bifidobacterium bifidum* TMC3115
Ctrl: Control group
Abx: Antibiotic exposure group
HFD: High-fat diet group
AHF: Antibiotic exposure + high-fat diet group
PE: Probiotic used in early life group
PW: Probiotic used throughout whole study group
PCoA: Principal coordinates analysis
PYY: Peptide YY
GLP-1: Glucagon-like peptide 1
GPR: G protein-coupled receptor
FXR: Farnesoid X receptor
TGR: G protein-coupled membrane receptor
