# Supplementary figures and images for: Dysbiosis of intestinal microbiota in early life aggravates high-fat diet induced dysmetabolism in adult mice
Source: BMC Microbiol. 2021 Jul 8;21:209. doi: 10.1186/s12866-021-02263-6 (PMC8268513; doi:10.1186/s12866-021-02263-6)

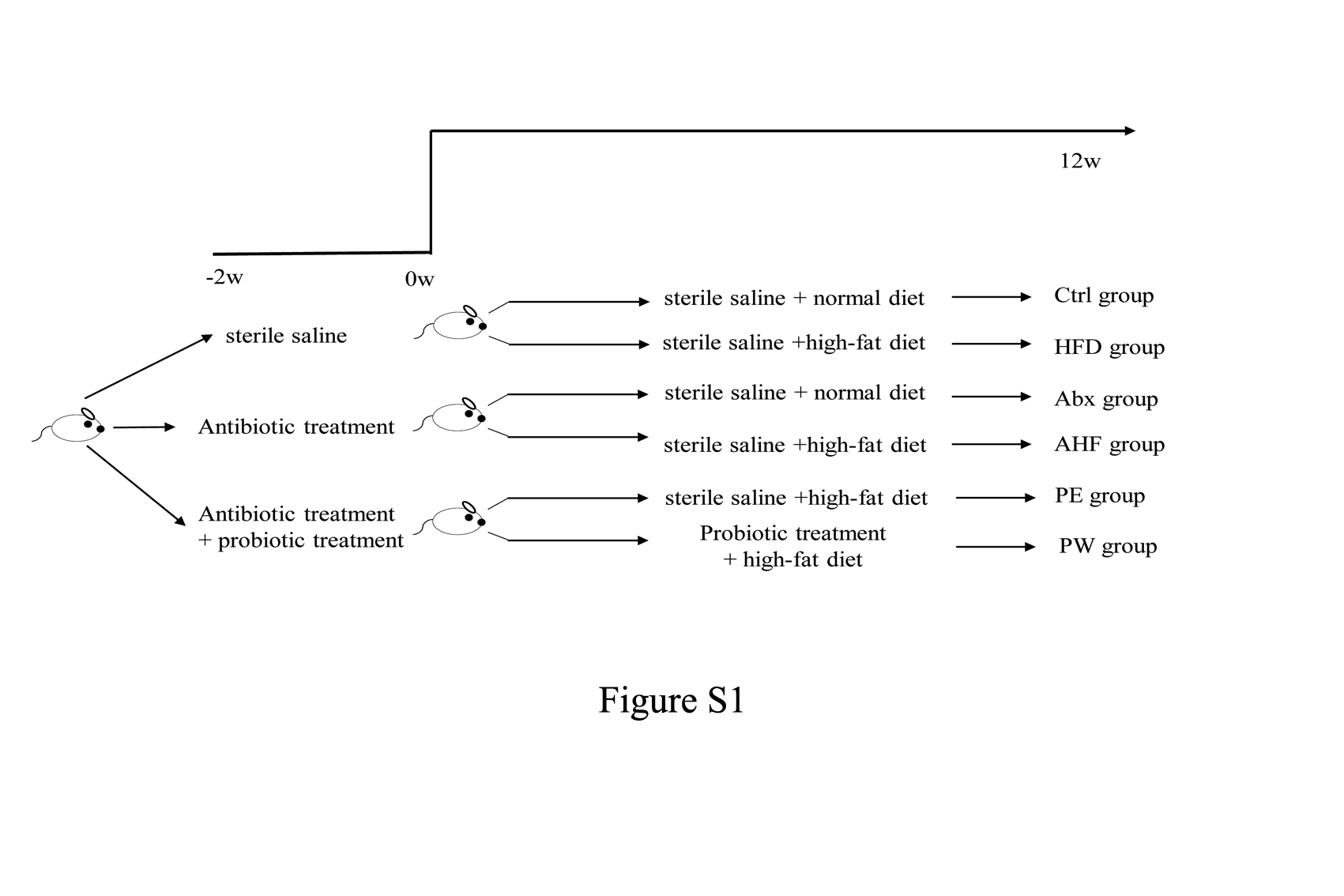

Supplement: Supplementary file 1 — Additional file 1. [file 12866_2021_2263_MOESM1_ESM.tif]
